# Comparative Expression Analysis of Olfactory Receptor Genes Among Individuals With Soldier and Worker Caste Differentiation Fates in Termites

**DOI:** 10.1002/ece3.72579

**Published:** 2025-12-11

**Authors:** Takumi Hanada, Masaru K. Hojo, Kiyoto Maekawa

**Affiliations:** ^1^ Graduate School of Science and Engineering University of Toyama Toyama Japan; ^2^ School of Biological and Environmental Sciences Kwansei Gakuin University Sanda Japan; ^3^ Faculty of Science, Academic Assembly University of Toyama Toyama Japan

**Keywords:** antennae, castes, chemoreception, RNA‐seq, termites

## Abstract

Clarifying the mechanisms controlling caste differentiation is a key research topic in the study of termite biology. The damp‐wood termite *Zootermopsis nevadensis* is the only species of termites in which soldier caste differentiation can be observed under natural conditions. In an incipient colony, soldier differentiation requires first‐molted 3rd‐instar larvae to engage in trophallactic behavior as a recipient from the reproductives. In contrast, other 3rd‐instar larvae exhibit worker‐like behaviors, such as allogrooming, and molt into later instars without undergoing soldier‐specific morphogenesis. Therefore, differences in behaviors between 3rd‐instar larvae with different caste differentiation fates are likely due to differences in chemosensory capacity for recognizing reproductives. To investigate this possibility, we focused on the expression patterns of olfactory genes in the main chemoreception organs (antennae) of the 3rd‐instar larvae in incipient colonies. We analyzed the duration from the first to the 4th‐instar and confirmed that the length of the 3rd‐instar period differed significantly between individuals with different developmental fates. RNA‐seq analysis of antennal tissues from 3rd‐instar larvae revealed that the olfactory receptor genes exhibiting significant expression differences between individuals with different caste differentiation fates were more frequently found in the *ionotropic receptors* (*IRs*) gene family (25/132 [18.9%]) than in the *odorant receptors* (*ORs*) gene family (6/62 [9.7%]). We paid particular attention to the gene expression differences of the co‐receptors (*Orco*, *IR8a* and *IR25a*), all of which are essential for odorant and ionotropic receptor functions. We performed real‐time quantitative PCR analysis using antennal tissues from individuals that had spent different numbers of days in the 3rd‐instar. The results showed that all co‐receptor genes were highly expressed in individuals with a worker differentiation fate from the very beginning of the 3rd‐instar. These results suggest that different chemosensory capacities among 3rd‐instar larvae are important for regulating caste differentiation fates in this species.

## Graphical abstract

We investigated the role of chemosensory gene expression in caste differentiation of the damp‐wood termite *Zootermopsis nevadensis* . Gene expression analyses revealed that third‐instar larvae destined to become workers exhibited significantly higher expression of key olfactory receptor genes in their antennae, suggesting that differences in chemosensory capacity underlie caste‐specific developmental pathways.

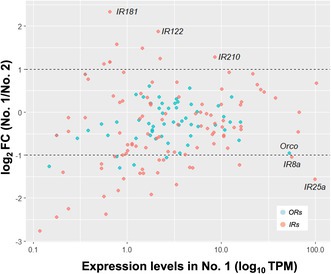

## 1 Introduction

Termites have castes that specialize in morphological and behavioral roles, enabling division of labor (Roisin 2000). Caste differentiation is tightly regulated in each colony, and the control mechanisms have long been the subject of research (Korb and Hartfelder 2008; Watanabe et al. 2014). Among the various caste differentiation factors, caste differentiation by olfactory substances such as pheromones has long been the focus of attention (Lefeuve and Bordereau 1984; Bordereau 1985; Noirot 1991). All insects, including termites, use chemical signaling as their primary communication tool (Greenfield 2002), and, in social insects, pheromone communication is considered an essential means of interaction between individuals (Wilson 1971; Noirot 1991). However, pheromones that regulate caste differentiation in termites have only been identified in a few species such as *Reticulitermes* spp. (Matsuura et al. 2010; Mitaka et al. 2016; Tarver et al. 2009). Consequently, the mechanisms by which these pheromonal substances are perceived and processed by colony members remain unclear.

In insects, olfactory molecules such as pheromones are received by olfactory receptors located on the dendrites of the olfactory nerve within the antennae (Wicher and Miazzi 2021). Olfactory receptors comprise two large gene families: *Odorant receptors* (*ORs*) and *Ionotropic receptors* (*IRs*) (Wicher and Miazzi 2021). ORs localized in olfactory sensory neurons (OSNs) in the antennae are involved in the reception of volatile molecules in *Drosophila melanogaster* (Kaupp 2010; Angioy et al. 2003). In contrast, IRs are responsible for the reception of amines and acidic molecules (Rytz et al. 2013) and are involved in the perception of humidity and taste in *D. melanogaster* (Rimal and Lee 2018). Interestingly, both these gene families have co‐receptor genes. *ORs* include an *Odorant receptor co‐receptor* (*Orco*) gene (Vosshall and Hansson 2011), and all ligand‐binding ORs function as selective ion channels to form complexes with Orco (Larsson et al. 2004; Neuhaus et al. 2005; Soffan et al. 2018). *IRs* have multiple co‐receptor genes, with *IR8a* and *IR25a* being highly conserved co‐receptor genes (Wicher and Miazzi 2021). Either IR8a or IR25a can form complexes with ligand‐binding IRs to function as nonselective cation channels (Benton et al. 2009; Abuin et al. 2011). The loss of co‐receptors in both ORs and IRs inhibits the function of all ligand‐binding ORs and IRs and significantly suppresses olfactory responses in *D. melanogaster* (DeGennaro et al. 2013; Abuin et al. 2011; Ai et al. 2013). Therefore, these co‐receptor genes are thought to be important for the insect olfactory system.

Olfactory receptors have been studied in termites and cockroaches. In the damp‐wood termite *Zootermopsis nevadensis* , whose genome was decoded for the first time in termites, 62 *ORs* and 132 *IRs* were identified (Terrapon et al. 2014). Furthermore, a large number of *IRs* have been identified in the drywood termite *Cryptotermes secundus* and the German cockroach *Blattella germanica* (Harrison et al. 2018). Molecular phylogenetic analyses suggest that the duplication of *IRs* occurs throughout Blattodea, including in termites (Harrison et al. 2018). In addition, comparisons of *ORs* in *B. germanica* and *Z. nevadensis* suggest that some genes have termite‐specific duplications (Robertson et al. 2018). The duplications of *ORs* have also been identified in eusocial hymenopterans (Zhou et al. 2012; Gadau et al. 2012). Moreover, the duplication of *OR* genes in the common ancestor of ants may have been crucial for the acquisition of sociality and the complexity of chemical signaling (Zhou et al. 2015). Indeed, in the ant *Temnothorax longispinosus*, the expression of *OR* genes in the antennae varied in a task‐dependent manner, highlighting the role of ORs in mediating the division of labor in colonies (Caminer et al. 2023). Species‐specific gene duplication of *ORs* may also be important for controlling social behavior in termites. Additionally, studies in several termite species have shown that *Orco* RNAi inhibits grooming and pheromone trail following behavior in individuals in the same colony (Sun et al. 2019; Gao et al. 2020; Lu et al. 2024; Xu et al. 2024). These previous studies focused on the role of ORs in short‐period behavior induced by releaser pheromones. However, the roles of olfactory receptors in physiological changes and caste differentiation remain unclear. To investigate the function of these olfactory genes, it is essential to focus on species in which caste differentiation can be easily observed under natural conditions.

*Zootermopsis nevadensis* is an important candidate for such analyses. In an incipient colony of this species, the first‐molted 3rd‐instar larva (hereafter called the No. 1 larva) has been observed to differentiate into a presoldier (intermediate stage of soldier differentiation), and the second‐molted 3rd‐instar larva (the No. 2 larva) differentiates into the 4th‐instar larva, which assumes the role of a worker in the colony (Maekawa et al. 2012). Third instar larvae that become soldiers (or workers) include both males and females. However, No. 1 larvae exhibited more frequent trophallactic behavior with reproductives (especially the queen) and a significantly shorter time to molt than did No. 2 larvae (Maekawa et al. 2012). Although this trophallactic behavior has been reported to be important for soldier differentiation (Yaguchi et al. 2018, 2025), it is not clear how such behavior is regulated between No. 1 larvae and reproductives. It is particularly interesting to investigate whether there is a difference in chemosensory capacity between No. 1 and No. 2 larvae, despite both being at the same 3rd‐instar developmental stage.

In the present study, we focused on the expression patterns of olfactory genes among individuals with different caste differentiation fates in *Z. nevadensis* . In previous studies, RNA‐seq analyses were performed using head tissues or whole bodies of No. 1 and No. 2 larvae (Masuoka et al. 2018; Yaguchi et al. 2018, 2019). However, because many olfactory genes are thought to be expressed in chemoreceptor organs, particularly in the antennae, it is possible that accurate expression patterns were not detected. Recently, transcriptome analyses have been performed using the antennae of various insect species, including termites, and different expression patterns of olfactory genes between sexes or developmental stages/castes have been reported (Koch et al. 2013; Chen et al. 2016; Suzuki et al. 2023; Raji et al. 2023; Liu et al. 2024; Lower et al. 2025). First, we measured the duration of each instar (from hatching to the 4th‐instar stage) to determine the most appropriate instar for comparative gene expression analysis. We then performed RNA‐seq and real‐time quantitative PCR (qPCR) analyses using the antennal tissues of No. 1 and No. 2 larvae. Based on these results, we discuss the differences in chemosensory ability between the larvae, in the context of their distinct caste developmental fates.

## 2 Results

### 2.1 Comparison of Duration of Each Instar Between Individuals With Different Caste Differentiation Fates

**FIGURE 1:**
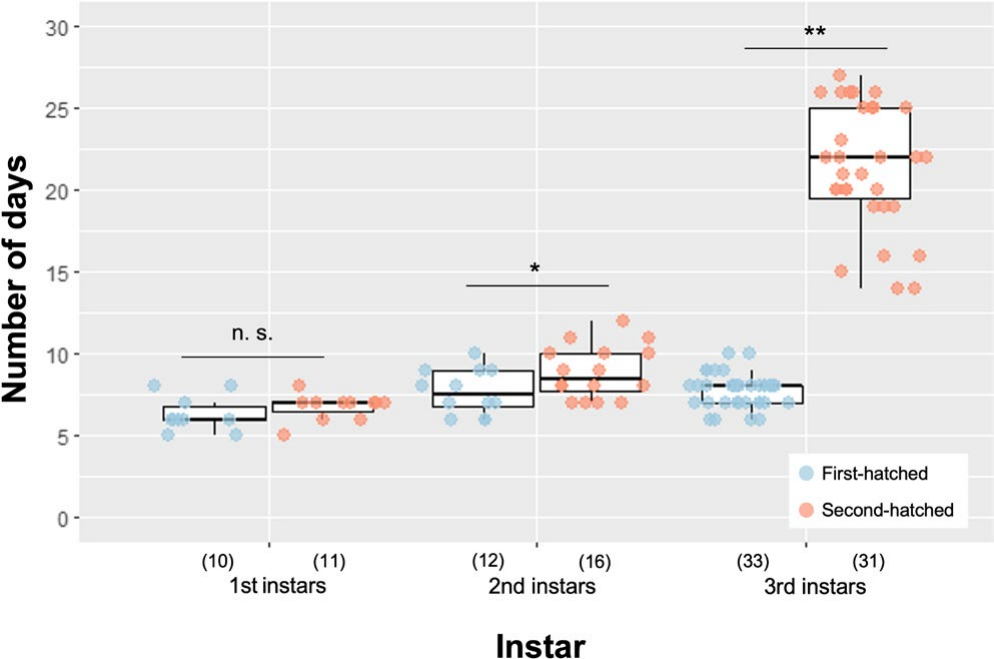
Duration of each instar of first‐hatched (blue) and second‐hatched (red) individuals in an incipient colony of *Zootermopsis nevadensis* . Asterisks indicate significant differences between first‐ and second‐hatched individuals (Welch's *t*‐test, * < 0.05, ** < 0.01).

In an incipient colony of *Z. nevadensis* , the first‐hatched individual is considered the first to molt and become a presoldier. To test this assumption, we measured the duration of each instar from hatching to the 4th‐instar stage in the first‐ and second‐hatched individuals (Table S1). The results showed no significant differences in the duration of the 1st instar stage (Welch's *t*‐test, *p* = 0.313), whereas the duration of the second‐instar stage was slightly shorter in first‐hatched individuals (Welch's *t*‐test, *p* = 0.043) (Figure 1). The duration of the 3rd‐instar stage was confirmed to be significantly shorter in first‐hatched individuals (No. 1 larvae) (Welch's *t*‐test, *p* < 0.001), as shown in a previous study (Maekawa et al. 2012). We excluded the results of four individuals (two first‐hatched and two second‐hatched) because their markings were accidentally removed, preventing us from tracking their differentiation fate. All other first‐hatched individuals differentiated into presoldiers, whereas the second‐hatched ones molted to 4th‐instar workers.

### 2.2 Expression Analysis of Olfactory Genes Between No. 1 and No. 2 Larvae

**FIGURE 2:**
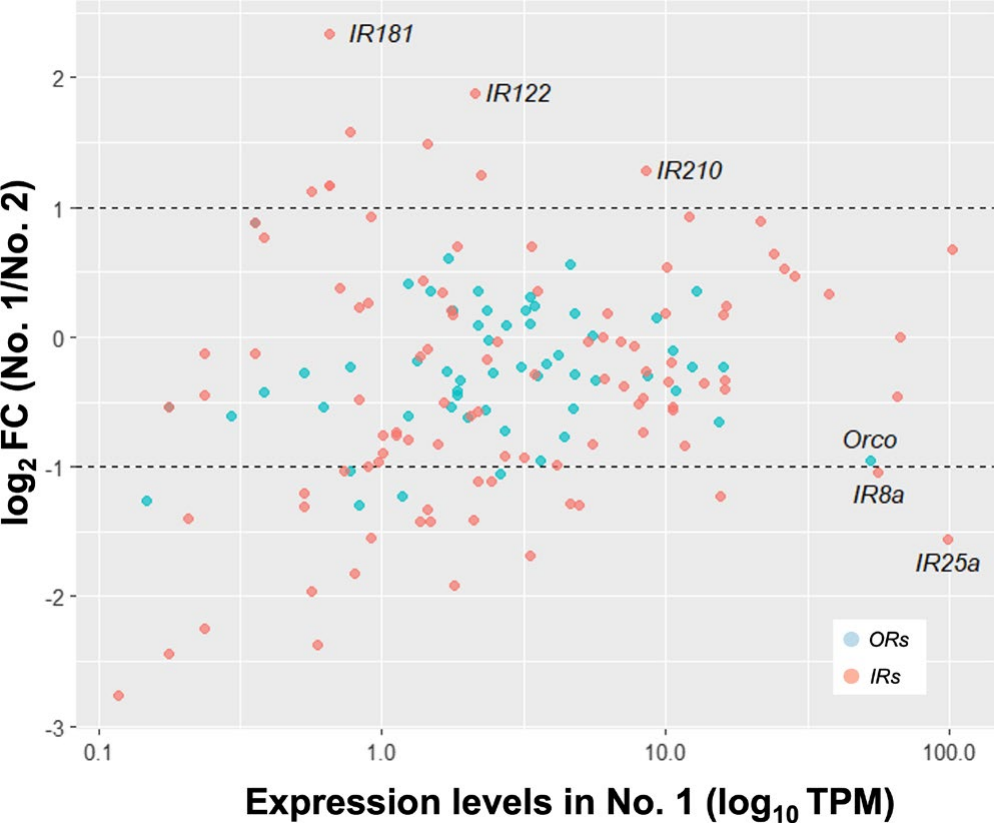
Antennal expression patterns of *Odorant receptors* (*ORs*) and *Ionotropic receptors* (*IRs*) in the first‐molted (No. 1 larvae) and second‐molted (No. 2 larvae) 3rd‐instar larvae. The *Y*‐axis represents the log_2_ fold change in gene expression in No. 1 larvae relative to No. 2 larvae. The *X*‐axis indicates the expression levels in No. 1 larvae (log_10_ TPM). *ORs* and *IRs* are shown in blue and red, respectively. The dashed lines indicate a twofold difference in expression.

RNA‐seq analysis was performed on the antennal tissues of No. 1 and No. 2 larvae at day 3 after molting. Of the 62 *OR* genes and 132 *IR* genes identified in the genome, 60 *ORs* and 123 *IRs* were expressed in the antennae during the 3rd‐instar stage. Twelve genes were uniquely expressed in either No. 1 or No. 2 larvae, but their expression levels were relatively low (transcripts per million [TPM] < 1, Tables S2 and S3). Among the 171 genes expressed in both larval types, 46 genes exhibited more than a twofold difference in expression (Figure 2, Figures S1 and S2). Most of these genes showing large fold‐change differences were *IRs* (41/113 [36.2%]), whereas only a small proportion were *ORs* (5/58 [8.6%]). Notably, the majority of the *IR* genes with large fold‐change differences were upregulated in No. 2 larvae (28/41 [68.2%]), and none of the *OR* genes showed higher expression in No. 1 larvae (0/5 [0%]). *IR181* and *IR122* were among the most strongly upregulated *IRs* in No. 1 larvae, whereas two co‐receptor genes (*IR8a* and *IR25a*) were upregulated in No. 2 larvae (Figure 2). Although *Orco* did not exceed the twofold change threshold, its expression trended higher in No. 2 larvae. A similar expression pattern was also observed in the differentially expressed gene (DEG) analysis. In the DEG analysis using a nonparametric statistical test, some *ORs* (6/62 [9.7%]) and *IRs* (25/132 [18.9%]) were detected as DEGs (Tables S2 and S3). All three co‐receptor genes (*Orco*, *IR8a*, and *IR25a*) were identified as DEGs and were highly expressed in No. 2 larvae. In contrast, *IR210* was the only DEG that showed higher expression in No. 1 larvae.

**FIGURE 3:**
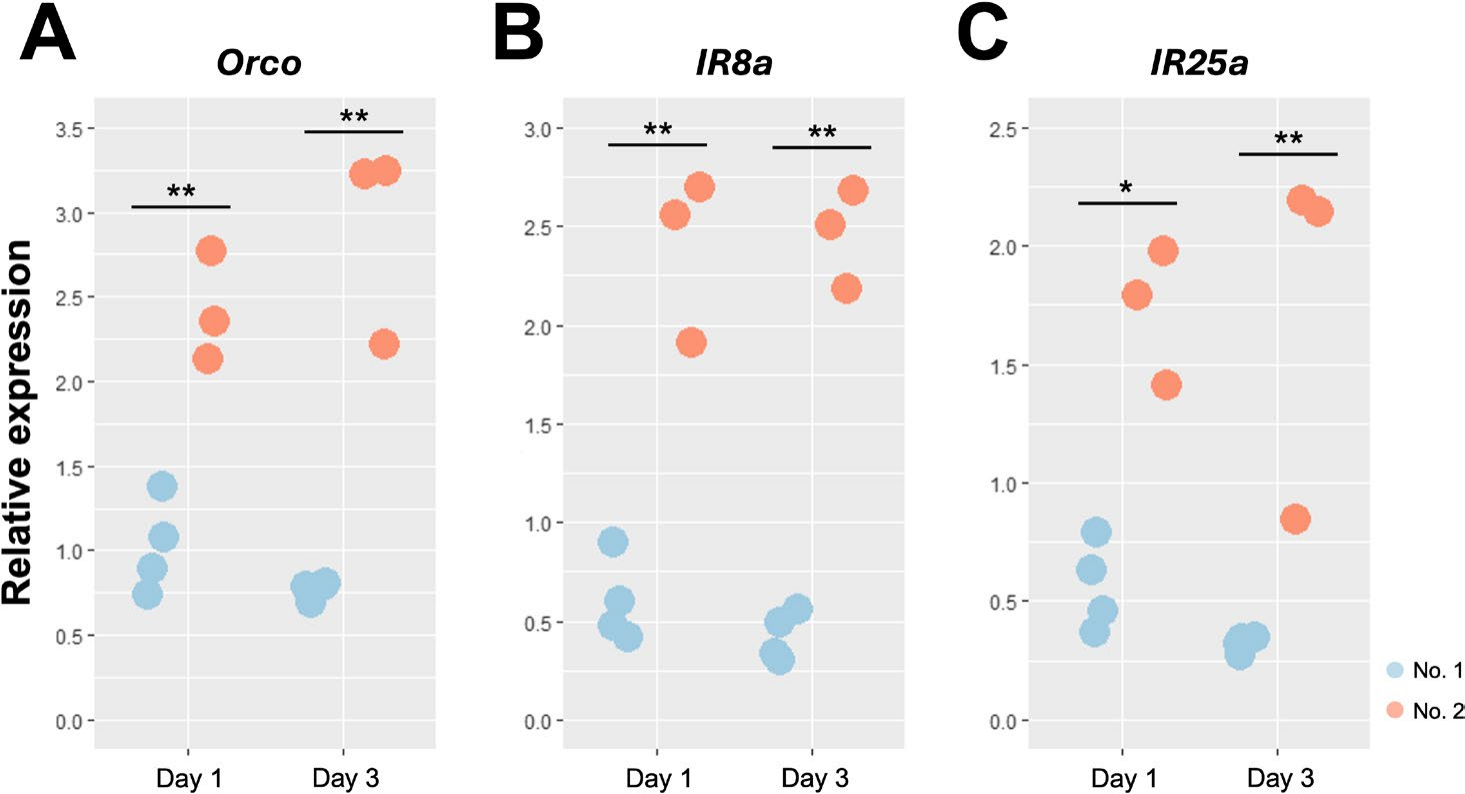
Antennal expression levels of *Orco*, *IR8a*, and *IR25a* in No. 1 and No. 2 larvae at day 1 and day 3 after molting (*n* = 3–4, mean ± SD). The numbers of samples examined are indicated in parentheses. Each value was normalized to the expression levels of *EF‐1alfa* (Table S4). Asterisks indicate significant differences between No. 1 and No. 2 larvae (Welch's *t*‐test, * < 0.05, ** < 0.01).

To further validate the differential expression patterns of the co‐receptor genes, we conducted real‐time qPCR analysis using antennal tissues from No. 1 and No. 2 larvae at days 1 and 3 after molting. Expression analysis confirmed that all three co‐receptor genes, *Orco*, *IR8a*, and *IR25a*, were significantly more highly expressed in No. 2 larvae at both days 1 and 3 (Figure 3).

## 3 Discussion

### 3.1 Behavioral Differences and Expression Patterns of Olfactory Genes Between No. 1 and No. 2 Larvae

We compared the expression patterns of olfactory receptor genes between 3rd‐instar larvae with different caste differentiation fates in *Z. nevadensis* . RNA‐seq analysis of antennal tissues from individuals destined to become soldiers (No. 1 larvae) and workers (No. 2 larvae) revealed distinct expression profiles, with most olfactory receptor genes showing higher expression levels in No. 2 larvae. In particular, three co‐receptor genes (*Orco*, *IR8a*, and *IR25a*) were significantly highly expressed in No. 2 larvae. These co‐receptor genes were highly expressed immediately after molting of No. 2 larvae. Among previous studies, Yaguchi et al. (2019) conducted a comprehensive gene expression analysis of 3rd‐instar larvae with biological replicates. Among the three co‐receptor genes focused on in this study, *Orco* and *IR8a* were identified as highly expressed DEGs in No. 2 larvae at day 3 after molting (Yaguchi et al. 2019). In addition, two *ORs* (*OR38* and *OR42*) and four *IRs* (*IR68a*, *IR75l*, *IR195*, and *IR202*) were detected as DEGs highly expressed in No. 1 larvae (Yaguchi et al. 2019), but these were not supported in the present study (Tables S2 and S3). These discrepancies are likely due to the use of whole‐body tissues in the previous study.

The elevated expression of the three co‐receptor genes in No. 2 larvae may indicate a generally enhanced chemosensory capacity compared to No. 1 larvae. Previous studies on the Formosan subterranean termite *Coptotermes formosanus* have shown that caste‐specific differences in antennal *Orco* expression are correlated with variations in olfactory sensitivity (Castillo et al. 2022). Similarly, in ants, *Orco* expression varies according to behavioral tasks, with higher *Orco* expression levels observed in nurse workers caring for broods (Caminer et al. 2023). In our study, No. 2 larvae also exhibit worker‐like behaviors, such as grooming other colony members (Maekawa et al. 2012). In contrast, No. 1 larvae do not engage in worker tasks but instead serve primarily as recipients of proctodeal trophallaxis from reproductives until presoldier differentiation (Maekawa et al. 2012). Consequently, the elevated *Orco* expression in No. 2 larvae likely reflects their broader behavioral repertoire.

Because significant differences in the expression of the three co‐receptor genes were already evident on the first day after molting (Figure 3), differences in chemosensory capacity may begin to develop during the early 3rd‐instar or even in the second‐instar. Indeed, we observed a significant difference in the duration of the second‐instar stage between the first‐ and second‐hatched larvae (Figure 1), suggesting that divergence in olfactory sensitivity may emerge early and influence subsequent behavioral differences. Nevertheless, when No. 1 larvae were removed from the colony within 24 h of molting, more than 70% of the colonies produced presoldiers from No. 2 larvae within 15 days (Maekawa et al. 2012), suggesting that the expression of co‐receptor genes and potentially other olfactory receptor genes remains plastic and is influenced by interactions with other individuals.

### 3.2 The Role of IRs in Regulating Behaviors That Promote Soldier Differentiation

The 3rd‐instar larvae may adjust their developmental fate in response to external stimuli. On day 3 of the 3rd‐instar is when reproductives begin frequent trophallactic interactions with No. 1 larvae (Maekawa et al. 2012; Yaguchi et al. 2018), suggesting that No. 1 larvae selectively perceive chemical signals derived from reproductives. Recent findings indicate that trail pheromones in termites are detected by specific ORs (Diallo et al. 2024), highlighting the role of ORs in pheromone communication. Interestingly, we found that all receptor genes that were highly expressed in No. 1 larvae were *IRs*, a gene family known to have undergone termite‐specific expansion (Harrison et al. 2018). Because IRs are involved in olfactory perception, their diversification may contribute to the complexity of termite chemical communication. This is supported by the caste‐ and sex‐specific expression patterns of IRs in termites revealed by RNA‐seq (using whole bodies without guts; Harrison et al. 2018). Among the *IRs* upregulated in No. 1 larvae, *IR181* and *IR122*, which exhibited particularly pronounced expression differences, and *IR210*, identified as a DEG, are promising candidates for mediating the perception of reproductive‐derived cues that potentially regulate soldier differentiation. In addition, No. 2 larvae spend more time performing allogrooming toward the queen than toward the king (Maekawa et al. 2012), indicating potential sex‐specific chemical interactions. Consequently, IRs are important candidates for involvement in the reception of sex‐specific signals from reproductives. Indeed, there are some reports of IR co‐receptor, which has been proposed to be involved in the reception of sex‐specific signals in other insect species. For example, in the leaf‐cutting ant *Atta vollenweideri* , *IR8a* and *IR25a* are highly expressed in adult males (Koch et al. 2013). Similarly, elevated *IR8a* expression has been observed in the male antennae of *Camponotus* and *Harpegnathos* ants (Zhou et al. 2012). In the German cockroach, *Blattella germanica* , *IR25a* is among the most highly expressed genes in adult male antennae (He et al. 2022). These findings imply that the observed differences in *IR* gene expression between 3rd‐instar larvae may be linked to the reception of sex‐specific olfactory signals, particularly those derived from the queen.

Insect chemoreceptor genes are expected to have low turnover rates, making it difficult to investigate the effects of gene expression changes on behavior (Kohlmeier and Billeter 2023). Indeed, a previous study using the American cockroach *Periplaneta americana* suggests that Orco protein turnover takes approximately one week (Tateishi et al. 2022). Although the turnover rates of ORs and IRs in *Z. nevadensis* remain unknown, if the turnover takes more than a week, suppression of gene expression must be maintained from stages earlier than the second instar. Because first‐ and second‐instar individuals are extremely fragile, we believe that genome editing methods, such as CRISPR/Cas9, are preferable for efficient functional analysis.

## 4 Experimental Procedures

### 4.1 Incipient Colony Foundation of *Z. nevadensis*

Mature colonies of the damp‐wood termite *Z. nevadensis* were collected from Kawanishi‐shi, Hyogo Prefecture, Japan, in April 2023. The collected colonies were placed in plastic containers and maintained in darkness at room temperature in the laboratory until the emergence of alates (winged adults). Alates were collected from at least five distinct mature colonies, and the sex of the individuals was confirmed using the morphology of the abdominal sternites (Weesner 1969). Incipient colonies were established as previously described (Maekawa et al. 2012; Yaguchi et al. 2018) by pairing female and male dealates in 60‐mm Petri dishes containing crushed wood chips. All incipient colonies were created by pairing dealates from different colonies. These dishes were kept stationary in an incubator at 25°C under constant dark conditions. A total of approximately 280 incipient colonies were used in the experiment.

### 4.2 Measurement of the Duration of Each Instar

We measured the duration of each instar from hatching to the 4th‐instar stage in the first‐ and second‐hatched individuals (Table S1). Each individual was marked on the abdomen with waterproof ink of different colors for identification. Colonies were checked every 24 h, and the time until the ink marks were lost due to molting was recorded. If the marking was removed during measurement, tracking was interrupted. No deaths of No. 1 (first‐hatched) and No. 2 (second‐hatched) individuals were observed in any of the incipient colonies used in this study. Statistical differences in the duration of each instar between first‐hatched and second‐hatched individuals were determined by Welch's *t*‐test using Mac Statistical Analysis ver. 3.0 (Esumi, Tokyo, Japan). The box plot and dot plot graphs were drawn using the “ggplot2” R package (Ginestet 2011).

### 4.3 Preparation for Antennal Tissues of No. 1 and No. 2 Larvae

We checked the incipient colonies every 24 h after confirming the presence of second‐instar larvae. No. 1 and No. 2 larvae were identified by marking the abdomen with waterproof ink of different colors. Each individual was collected on day 1 (for real‐time qPCR) and day 3 (for real‐time qPCR and RNA‐seq) after molting. For each sample, antennal tissues were cut from 10 (for real‐time qPCR) or 50 individuals (for RNA‐seq). All 3rd‐instar larvae (No. 1 and No. 2) used for RNA extraction were separately collected from 1 individual per colony. Each sample was immediately frozen in liquid nitrogen and then stored at −80°C.

### 4.4 RNA‐Seq Analysis

Total RNA was extracted from the antennal tissues (50 individuals for each sample) of No. 1 and No. 2 larvae using the ReliaPrep RNA Tissue Miniprep System (Promega, Madison, WI, USA), and DNase treatment was performed using the same kit. The amounts of RNA and DNA in each sample were quantified using a Qubit 2.0 fluorometer (Life Technology, Eugene, OR, USA). Directional mRNA library preparation and sequencing (total 86,606,132 reads (No. 1 larva), 72,441,092 reads (No. 2 larva)) were performed by Novogene Co. Ltd. (Tianjin, China) using NovaSeq at 150 bp paired‐end (PE) reads. The adapter sequences, low quality (< q30), and short sequences (< 50 bp) were removed using fastp v0.20.1 (Chen et al. 2018). The cleaned reads were mapped to the *Z. nevadensis* reference genome (gene model Znev.OGS.v2.2; Terrapon et al. 2014) using HISAT2 v2.1.0 (Kim et al. 2015) with default parameters. Reads were counted using featureCounts v2.0.3 (Liao et al. 2014). Identification of DEGs was performed by nonparametric statistical analysis using “NOISeq” R package (Tarazona et al. 2011, 2015). The counted reads were corrected for TPM and the expression levels of each olfactory receptor gene were analyzed. Scatter plots of *ORs* and *IRs* were drawn using the “ggplot2” R package (Ginestet 2011). Heatmaps of *ORs* and *IRs* were drawn using the “pheatmap” R package (Kolde 2025), and genes exhibiting similar expression patterns among 3rd‐instar larvae were clustered. All RNA‐seq reads were deposited in the DDBJ Sequence Read Archive database under BioProject accession number PRJDB35510.

### 4.5 Real‐Time qPCR Analysis

Expression levels of genes other than the co‐receptors of *ORs* and *IRs* were relatively low (Tables S2 and S3). Because the number of individuals available for collecting antennal tissues from No. 1 and No. 2 larvae was limited, it was challenging to obtain sufficient cDNAs to accurately quantify the expression levels of genes with low expression. Therefore, it was necessary to select the genes to be analyzed by real‐time quantitative PCR. We focused on the co‐receptors of *ORs* and *IRs*, which are known to be functionally important (DeGennaro et al. 2013; Abuin et al. 2011; Ai et al. 2013). Total RNA was extracted from antennal tissues (10 individuals per sample) on days 1 and 3 after the molting of No. 1 and No. 2 larvae. Total RNA extraction and DNase treatment were performed using a ReliaPrep RNA Tissue Miniprep System (Promega). RNA and DNA concentrations were quantified using a Qubit 2.0 fluorometer (Life Technology). The RNA purity and quantity were determined by spectroscopic measurements at 230, 260, and 280 nm using a NanoVue spectrophotometer (GE Healthcare Bio‐Sciences, Tokyo, Japan). Single‐stranded cDNA was synthesized from equal quantities of DNase‐treated RNA (180 ng) using a High‐Capacity cDNA Reverse Transcription Kit (Thermo Fisher Scientific, Waltham, MA, USA). Relative quantification of transcripts was performed using the Thunderbird SYBR qPCR Mix (Toyobo) and QuantStudio 3 Real‐Time PCR System (Thermo Fisher Scientific). According to previous studies (Masuoka et al. 2015; Yaguchi et al. 2018), the suitability of six reference genes, *beta‐actin* (GenBank accession no. AB915826), *EF‐1alfa* (AB915828), *NADH‐dh* (AB936819), *RS49* [geneID: *KDR21989* (*Znev_08151*)], *RPS18* [*KDR2265*1 (*Znev_00110*)], and *RPL13a* [*KDR22610* (*Znev_00068*)] was evaluated using GeNorm (Vandesompele et al. 2002) and NormFinder (Andersen et al. 2004) software. Based on the obtained stability values, we selected *EF1‐alfa* as an internal control gene (Table S4). We designed the qRT‐PCR primers for all three target genes using Primer3Plus (Untergasser et al. 2007; Table S5). Amplification efficiencies were obtained separately for the target and reference genes, and standardized target amounts and relative expression levels were calculated. Statistical analysis was performed using Welch's *t*‐test to examine the differences in expression levels between No. 1 and No. 2 larvae using Mac Statistical Analysis ver. 3.0 (Esumi). The dot plot graphs of expression levels were drawn using the “ggplot2” R package (Ginestet 2011).

## Author Contributions

**Takumi Hanada:** conceptualization (equal), data curation (equal), formal analysis (equal), funding acquisition (equal), investigation (equal), visualization (equal), writing – original draft (equal), writing – review and editing (equal). **Masaru K. Hojo:** conceptualization (equal), data curation (equal), formal analysis (equal), validation (equal), visualization (equal), writing – original draft (equal), writing – review and editing (equal). **Kiyoto Maekawa:** conceptualization (equal), data curation (equal), formal analysis (equal), funding acquisition (equal), investigation (equal), project administration (lead), supervision (lead), validation (equal), visualization (equal), writing – original draft (equal), writing – review and editing (equal).

## Funding

This study was supported by Japan Society for the Promotion of Science, Grant/Award Number: JP23K23935 and JP25K02326; Japan Science and Technology Agency SPRING, Grant/Award Number: JPMJSP2145.

## Conflicts of Interest

The authors declare no conflicts of interest.

## Supporting information


**Figure S1:** Antennal expression patterns of *Odorant receptors* (*ORs*) between No. 1 and No. 2 larvae. The colors show the log1p‐transformed TPM values of each gene, with red and blue indicating high and low expression, respectively. Genes exhibiting similar expression patterns among 3rd‐instar larvae were clustered. The red circles indicate genes with more than a twofold higher expression in No. 2 compared to No. 1 larvae.
**Figure S2:** Antennal expression patterns of *Ionotropic receptors* (*IRs*) between No. 1 and No. 2 larvae. The colors show the log1p‐transformed TPM values of each gene, with red and blue indicating high and low expression, respectively. Genes exhibiting similar expression patterns among 3rd‐instar larvae were clustered. Blue and red circles indicate genes with more than a twofold higher expression in No. 1 and No. 2 larvae, respectively.

**Table S1:** Number of days for each instar.
**Table S2:** Expression levels of Odorant receptor (OR) genes of No. 1 and No. 2 larvae.
**Table S3:** Expression levels of Ionotropic receptor (IR) genes of No. 1 and No. 2 larvae.
**Table S4:** Stability values of reference genes in real‐time qPCR analysis.
**Table S5:** Primer sequence used for real‐time qPCR analysis.

## Data Availability

RNA‐seq data obtained are available from the DDBJ Sequence Read Archive database under BioProject accession number PRJDB35510. All other relevant data is within the paper and its Supporting Information.
