# Supplementary material for: Comparative Expression Analysis of Olfactory Receptor Genes Among Individuals With Soldier and Worker Caste Differentiation Fates in Termites
Source: Ecol Evol. 2025 Dec 11;15(12):e72579. doi: 10.1002/ece3.72579 (PMC12698947; doi:10.1002/ece3.72579)
Supplement: Supplementary file 1 — Figure S1: Antennal expression patterns of Odorant receptors (ORs) between No. 1 and No. 2 larvae. The colors show the log1p‐transformed TPM values of each gene, with red and blue indicating high and low expression, respectively. Genes exhibiting similar expression patterns among 3rd‐instar larvae were clustered. The red circles indicate genes with more than a twofold higher expression in No. 2 compared to No. 1 larvae. Figure S2: Antennal expression patterns of Ionotropic receptors (IRs) between No. 1 and No. 2 larvae. The colors show the log1p‐transformed TPM values of each gene, with red and blue indicating high and low expression, respectively. Genes exhibiting similar expression patterns among 3rd‐instar larvae were clustered. Blue and red circles indicate genes with more than a twofold higher expression in No. 1 and No. 2 larvae, respectively. [file ECE3-15-e72579-s002.pdf]

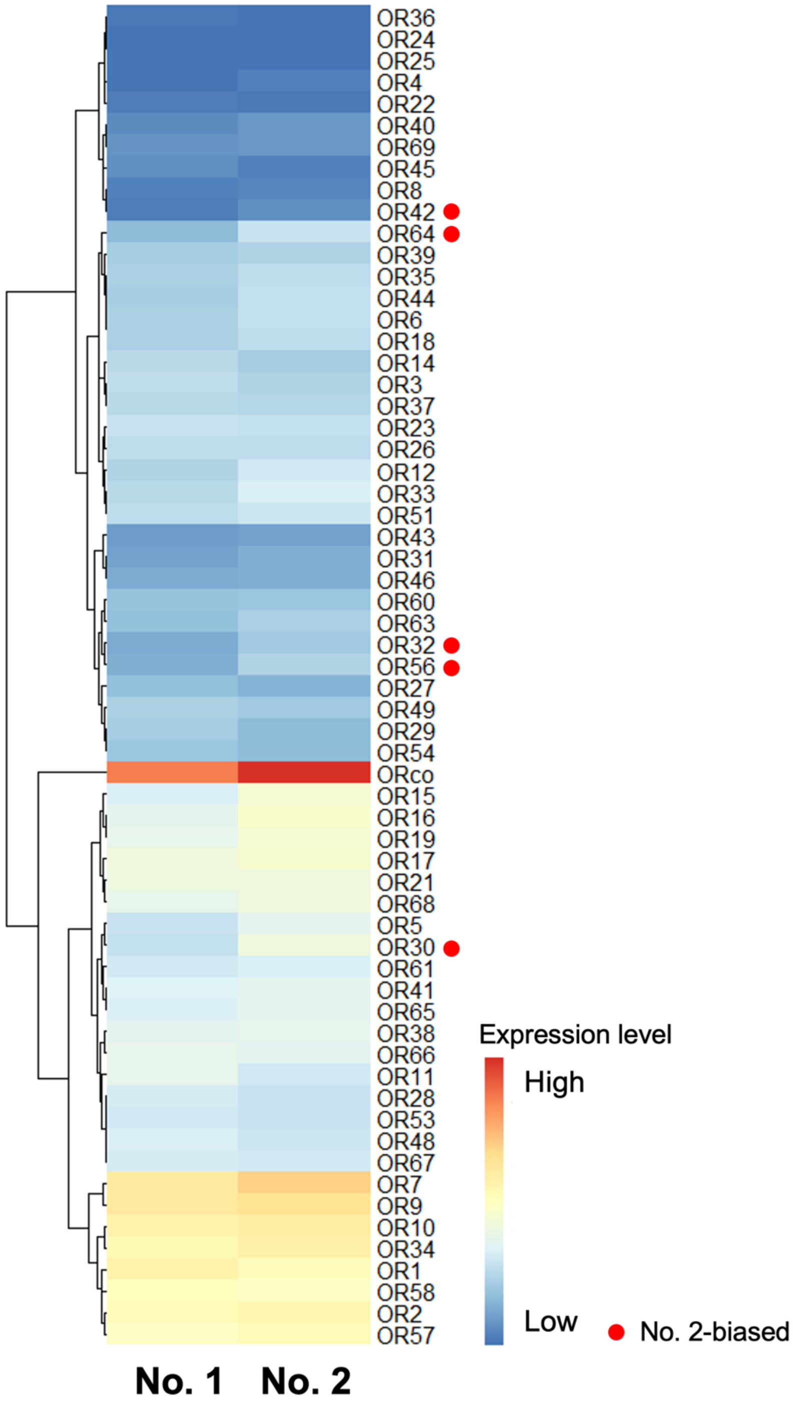

Supplementary Fig. 1

Antennal expression patterns of *Odorant receptors* (*ORs*) between No. 1 and No. 2 larvae. The colors show the log1p-transformed TPM values of each gene, with red and blue indicating high and low expression, respectively. Genes exhibiting similar expression patterns among 3rd-instar larvae were clustered. The red circles indicate genes with more than a two-fold higher expression in No. 2 compared to No. 1 larvae.

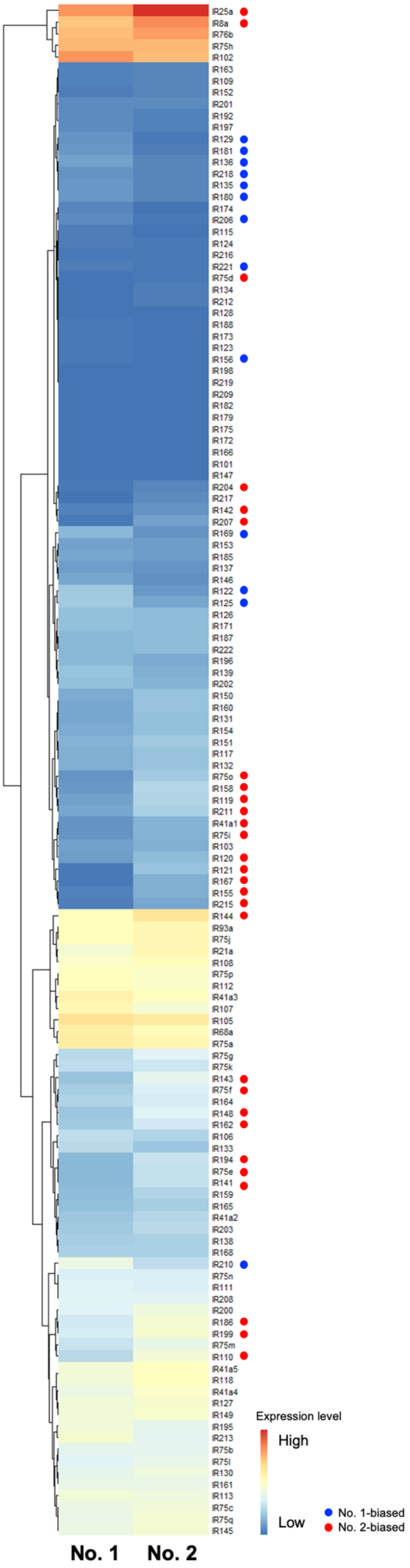

Supplementary Fig. 2

Antennal expression patterns of *Ionotropic receptors* (*IRs*) between No. 1 and No. 2 larvae. The colors show the log1p-transformed TPM values of each gene, with red and blue indicating high and low expression, respectively. Genes exhibiting similar expression patterns among 3rd-instar larvae were clustered. Blue and red circles indicate genes with more than a two-fold higher expression in No. 1 and No. 2 larvae, respectively.
