# Supplementary material for: Comparative Expression Analysis of Olfactory Receptor Genes Among Individuals With Soldier and Worker Caste Differentiation Fates in Termites
Source: Ecol Evol. 2025 Dec 11;15(12):e72579. doi: 10.1002/ece3.72579 (PMC12698947; doi:10.1002/ece3.72579)
Supplement: Supplementary file 2 — Table S1: Number of days for each instar. Table S2: Expression levels of Odorant receptor (OR) genes of No. 1 and No. 2 larvae. Table S3: Expression levels of Ionotropic receptor (IR) genes of No. 1 and No. 2 larvae. Table S4: Stability values of reference genes in real‐time qPCR analysis. Table S5: Primer sequence used for real‐time qPCR analysis. [file ECE3-15-e72579-s001.pdf]

Table S1. Number of days for each instar

1-instar		2-instar		3-instar	
First-molted	Second-molted	First-molted	Second-molted	First-molted	Second-molted
7	7	7	11	8	25
6	7	7	7	9	25
6	7	6	8	8	22
5	8	6	8	7	26
8	7	7	10	7	19
5	7	9	7	9	20
6	6	8	8	6	20
6	7	8	7	8	22
8	5	10	10	7	19
6	6	6	8	6	21
	7	9	7	8	26
		9	9	7	20
			11	7	22
			12	8	22
			10	8	26
			9	6	16
				8	21
				7	20
				6	25
				8	25
				8	19
				7	22
				8	27
				7	26
				7	23
				8	20
				7	26
				8	14
				10	16
				9	15
				9	14
				8	
				10	

Table S2. Expression levels of odorant receptor (OR) genes of No. 1 and No. 2 larvae

Gene name[1]	Gene ID[1]	Expression level (TPM) of No. 1 larvae	Expression level (TPM) of No. 2 larvae	Probability[2]
Orco	Znev_11756	52.57846197	101.6249403	***0.953085896***
OR1	Znev_18978	12.7453977	9.975849113	0.606496995
OR2	Znev_18979	10.52752107	11.32741577	0.744185224
OR3	Znev_18973	2.336163383	2.027349981	0.052923294
OR4	Znev_18894	0	0.193080951	0.40330152
OR5	Znev_18893	2.720595332	4.473042022	0.887274655
OR6	Znev_18892	1.833444681	2.510052357	0.709498056
OR7	Znev_18891	15.31813459	24.16729898	***0.911990103***
OR8	Znev_18890	0.17743013	0.257441267	0.223619654
OR9	Znev_19033	15.99828342	18.82539268	0.835542595
OR10	Znev_18846	12.39053744	14.48107129	0.827511488
OR11	Znev_18824	4.61318339	3.121475368	0.678801697
OR12	Znev_07294	2.010874811	3.089295209	0.812866737
OR14	Znev_19012	2.188304941	1.705548397	0.284616472
OR15	Znev_19013	3.607745984	6.983094379	***0.946553552***
OR16	Znev_18852	4.376609883	7.465796755	***0.923220219***
OR17	Znev_18908	5.677764172	7.143995171	0.832965712
OR18	Znev_18907	1.833444681	2.445692041	0.684538706
OR19	Znev_18906	4.731470143	6.950914221	0.883145988
OR21	Znev_18983	5.52990573	5.470626933	0.435383528
OR22	Znev_18982	0.147858442	0.096540475	0.111788618
OR23	Znev_18981	2.750167021	2.574412674	0.156903499
OR24	Znev_18948	0	0	-
OR25	Znev_18947	0	0	-
OR26	Znev_18988	2.365735072	2.413511882	0.34845175
OR27	Znev_18841	1.242010913	0.933224594	0.254227642
OR28	Znev_18823	3.3120291	2.67095315	0.277292329
OR29	Znev_19044	1.715157927	1.126305545	0.462078473
OR30	Znev_19008	2.602308579	5.406266616	***0.951028632***
OR31	Znev_19009	0.621005456	0.901044436	0.471343231
OR32	Znev_19010	0.768863898	1.576827763	0.786344998
OR33	Znev_18839	2.306591695	3.411096793	0.814980559
OR34	Znev_19038	10.79366626	14.3201705	0.875295157
OR35	Znev_19021	1.892588057	2.381331724	0.630289855
OR36	Znev_19027	0.088715065	0	0.215500177
OR37	Znev_18975	2.188304941	2.059530139	0.151785083
OR38	Znev_18972	4.169608064	4.601762655	0.639236479
OR39	Znev_18971	1.685586239	2.027349981	0.542739484
OR40	Znev_18970	0.295716884	0.450522218	0.334733121
OR41	Znev_18870	3.785176115	4.376501546	0.689042064
OR42	Znev_18871	0.147858442	0.353981743	0.42340403
OR43	Znev_18872	0.532290391	0.643603169	0.290137858
OR44	Znev_18873	1.744729615	2.542232516	0.74903853
OR45	Znev_18874	0.354860261	0.193080951	0.251276069
OR46	Znev_18875	0.768863898	0.901044436	0.328147755
OR48	Znev_18838	3.459887542	2.928394417	0.148317427
OR49	Znev_18836	1.774301304	1.544647605	0.040144928
OR51	Znev_18837	2.454450137	2.960574575	0.64640509
OR53	Znev_18855	3.193742347	2.767493625	0.067136797

OR54	Znev_19048	1.47858442	1.158485703	0.222820785
OR56	Znev_19036	0.828007275	2.027349981	0.874340756
OR57	Znev_19035	8.605361323	10.58727212	0.842400141
OR58	Znev_19034	9.285510157	8.366841191	0.082852598
OR60	Znev_19068	1.330725978	1.512467446	0.407239307
OR61	Znev_19069	3.075455593	3.604177744	0.660512549
OR63	Znev_19061	1.242010913	1.898629347	0.702082008
OR64	Znev_19062	1.182867536	2.767493625	***0.915217391***
OR65	Znev_19063	3.519030919	4.312141229	0.746242489
OR66	Znev_19064	4.761041832	4.183420596	0.02941322
OR67	Znev_19065	3.3120291	3.089295209	0.159579357
OR68	Znev_19056	4.79061352	5.856788834	0.792764228
OR69	Znev_19070	0.384431949	0.514882535	0.306306115

[1]Terrapon et al., 2014

[2]Bold blue italic numbers indicate the significant differences caluculated by NOISeq (probability > 0.9).

Table S3. Expression levels of ionotropic receptor (IR) genes of No. 1 and No. 2 larvae

Gene name[1]	Gene ID[1]	Expression level (TPM) of No. 1 larvae	Expression level (TPM) of No. 2 larvae	Probability[2]
IR25a	Znev_19054	98.50329405	290.6511909	***0.976935313***
IR8a	Znev_18858	56.0679212	115.3980481	***0.962371863***
IR93a	Znev_18953	16.08699849	21.30326488	0.877603393
IR76b	Znev_18807	65.32385967	90.1044436	0.888080594
IR68a	Znev_18905	26.02308579	18.02088872	0.81727819
IR21a	Znev_18951	11.71038861	20.91710298	***0.936730293***
IR41a1	Znev_19017	0.532290391	1.22284602	0.74602333
IR41a2	Znev_18835	2.040446499	3.121475368	0.813732768
IR41a3	Znev_18867	23.86435254	15.31775541	0.853708024
IR41a4	Znev_18862	8.309644439	13.77310781	***0.921117002***
IR41a5	Znev_18861	10.58666445	15.67173715	0.898784023
IR75a	Znev_18960	28.5366793	20.59530139	0.787956875
IR75b	Znev_18959	6.890203396	7.079634854	0.58845175
IR75c	Znev_18958	8.546217947	10.2976507	0.830933192
IR75d	Znev_18957	0.029571688	0.064360317	0.156334394
IR75e	Znev_18956	1.360297666	3.636357902	***0.950922587***
IR75f	Znev_18955	2.424878449	5.245365824	***0.952863203***
IR75g	Znev_18954	3.164170658	6.017689626	***0.939946978***
IR75h	Znev_19067	67.21644773	67.09563032	0.662255214
IR75i	Znev_18989	0.532290391	1.319386496	0.781286674
IR75j	Znev_19005	16.20528524	20.4344006	0.86310357
IR75k	Znev_18967	3.459887542	4.215600754	0.736376812
IR75l	Znev_18968	6.09176781	7.594517389	0.831502298
IR75m	Znev_18809	4.110464687	8.141580082	***0.952993991***
IR75n	Znev_18810	5.322903911	5.438446774	0.527320608
IR75o	Znev_18811	0.56186208	2.188250773	***0.934878049***
IR75p	Znev_18812	15.87999667	14.15926971	0.051311417
IR75q	Znev_18813	7.984355867	11.42395624	0.890509014
IR101	Znev_18847	0	0	-
IR102	Znev_19025	102.4363286	64.16723591	0.862990456
IR103	Znev_19011	0.828007275	1.158485703	0.516868151
IR105	Znev_19059	37.58561595	29.9597275	0.619890421
IR106	Znev_18923	3.548602608	2.767493625	0.370996819
IR107	Znev_19049	21.46904578	11.52049672	0.897462001
IR108	Znev_18799	13.60297666	17.44164587	0.867143867
IR109	Znev_18819	0.236573507	0.257441267	0.12969954
IR110	Znev_18820	3.3120291	10.65163244	***0.979777306***
IR111	Znev_18922	6.210054563	5.470626933	0.027334747
IR112	Znev_18920	16.23485693	13.74092765	0.311640156
IR113	Znev_18919	9.96565899	8.753003093	0.047423118
IR115	Znev_18918	0.17743013	0.032180158	0.323517144
IR117	Znev_18917	1.123724159	1.898629347	0.753361612
IR118	Znev_18933	10.55709276	15.34993557	0.895599152
IR119	Znev_18934	0.798435587	2.831853942	***0.953708024***
IR120	Znev_18935	0.73929221	1.512467446	0.775478968
IR121	Znev_18936	0.088715065	1.866449189	***0.952371863***
IR122	Znev_18937	2.129161565	0.579242852	0.882916225
IR123	Znev_03024	0.059143377	0	0.15115942
IR124	Znev_18938	0.147858442	0.096540475	0.111788618

IR125	Znev_18939	2.21787663	0.933224594	0.8056345
IR126	Znev_18940	1.744729615	1.512467446	0.045461294
IR127	Znev_18941	10.40923432	11.93883878	0.807712973
IR128	Znev_18883	0.029571688	0	0.06572994
IR129	Znev_18884	0.532290391	0.064360317	0.60630258
IR130	Znev_18885	7.126776904	9.235705469	0.860572641
IR131	Znev_18886	0.887150652	1.769908714	0.805998586
IR132	Znev_18887	1.123724159	1.866449189	0.7421421
IR133	Znev_18888	3.341600789	2.059530139	0.695768823
IR134	Znev_18830	0	0.128720634	0.313934252
IR135	Znev_18829	0.650577145	0.289621426	0.454906327
IR136	Znev_18828	0.768863898	0.257441267	0.587992223
IR137	Znev_19018	0.709720522	0.547062693	0.162407211
IR138	Znev_19019	2.336163383	2.638772991	0.535705196
IR139	Znev_19020	1.833444681	1.126305545	0.542156239
IR141	Znev_18943	1.449012731	3.636357902	***0.946458112***
IR142	Znev_18915	0.207001819	0.547062693	0.551180629
IR143	Znev_18825	1.803872992	6.790013428	***0.985517851***
IR144	Znev_18826	15.46599303	36.23485839	***0.967408978***
IR145	Znev_18859	8.339216128	11.52049672	0.882955108
IR146	Znev_18834	0.91672234	0.482702376	0.458398727
IR147	Znev_10307	0	0	-
IR148	Znev_19050	2.099589876	5.599347567	***0.966380346***
IR149	Znev_18946	10.17266081	12.90424353	0.861435136
IR150	Znev_19006	1.005437405	1.866449189	0.792672322
IR151	Znev_19007	1.242010913	2.156070615	0.799597031
IR152	Znev_18890	0.17743013	0.257441267	0.223619654
IR153	Znev_19030	0.828007275	0.707963485	0.050130788
IR154	Znev_19031	1.005437405	1.705548397	0.729561683
IR155	Znev_19032	0.236573507	1.126305545	0.823835277
IR156	Znev_19045	0.059143377	0	0.15115942
IR158	Znev_18817	0.591433768	3.057115051	***0.974326617***
IR159	Znev_18986	1.567299485	2.767493625	0.852598091
IR160	Znev_18987	0.975865717	1.898629347	0.812234005
IR161	Znev_19028	7.74778236	8.109399924	0.657819017
IR162	Znev_18800	2.188304941	4.730483289	***0.949091552***
IR163	Znev_18806	0.236573507	0.321801584	0.234920467
IR164	Znev_18930	2.691023644	5.084465032	***0.932636974***
IR165	Znev_18961	1.65601455	2.349151565	0.715044185
IR166	Znev_18962	0	0	-
IR167	Znev_18963	0.059143377	1.190665862	0.880063627
IR168	Znev_18833	2.543165202	2.606592833	0.373312124
IR169	Znev_18977	1.449012731	0.514882535	0.750625663
IR171	Znev_18866	1.774301304	1.576827763	0.005224461
IR172	Znev_19058	0	0	-
IR173	Znev_18952	0.059143377	0	0.15115942
IR174	Znev_18999	0.295716884	0	0.47543655
IR175	Znev_18827	0	0	-
IR179	Znev_16376	0	0	-
IR180	Znev_18976	0.650577145	0.289621426	0.454906327
IR181	Znev_18831	0.650577145	0.128720634	0.625461294
IR182	Znev_18832	0	0	-

IR185	Znev_19037	0.887150652	0.740143644	0.087525627
IR186	Znev_18944	4.583611701	11.19869513	***0.968176034***
IR187	Znev_18945	1.360297666	1.512467446	0.382209261
IR188	Znev_19004	0.059143377	0	0.15115942
IR192	Znev_19053	0.354860261	0.193080951	0.251276069
IR194	Znev_19057	1.47858442	3.958159487	***0.955606221***
IR195	Znev_18984	10.02480237	6.918734062	0.782403676
IR196	Znev_18904	1.389869355	1.02976507	0.287398374
IR197	Znev_19003	0.384431949	0.225261109	0.237889714
IR198	Znev_18950	0	0.032180158	0.106581831
IR199	Znev_18856	4.938471962	12.13191973	***0.968706257***
IR200	Znev_18857	5.52990573	9.814948321	***0.933746907***
IR201	Znev_18854	0.354860261	0.386161901	0.16204666
IR202	Znev_18942	1.626442862	1.287206337	0.214351361
IR203	Znev_19066	2.188304941	3.250196001	0.808292683
IR204	Znev_18853	0.059143377	0.321801584	0.493764581
IR206	Znev_19014	0.354860261	0.064360317	0.471519972
IR207	Znev_18974	0.118286754	0.804503961	0.758038176
IR208	Znev_19055	6.003052745	5.985509468	0.476620714
IR209	Znev_18863	0	0	-
IR210	Znev_03026	8.516646258	3.507637269	***0.946355603***
IR211	Znev_19060	0.91672234	2.67095315	***0.932997526***
IR212	Znev_18985	0	0.128720634	0.313934252
IR213	Znev_18895	12.03567718	6.33949121	0.89659597
IR215	Znev_19041	0.17743013	0.965404753	0.793997879
IR216	Znev_19042	0.118286754	0.064360317	0.127649346
IR217	Znev_18845	0	0.386161901	0.584722517
IR218	Znev_18844	0.56186208	0.257441267	0.407712973
IR219	Znev_18814	0	0	-
IR221	Znev_18843	0.147858442	0.064360317	0.196440438
IR222	Znev_18889	1.449012731	1.544647605	0.334817957

[1]Terrapon et al., 2014

[2]Bold blue italic numbers indicate the significant differences caluculated by NOISeq (probability > 0.9).

Table S4. Stability values of reference genes in real-time qPCR analysis

Gene name	Gene ID/Accession no[1]	Stability value GeNorm[2]	Stability value NormFinder[3]
Znbeta-actin	AB915826	1.312	0.893
ZnEF-1a*	AB915828	0.466	0.032
ZnNADH-dh	AB936819	0.566	0.159
ZnRS49	KDR21989 (Znev_08151)	0.487	0.032
ZnRPS18	KDR22651 (Znev_00110)	0.853	0.534
ZnRPL13a	KDR22610 (Znev_00068)	0.474	0.051

[1]Gene ID of *Zootermopsis nevadensis* (Terrapone et al. 2014) or DDBJ/EMBL/GenBank Accession No.

[2]Vandesompele et al., 2002

[3]Andersen et al., 2004

*ZnEF-1a was selected by GeNorm and NormFinder due to the lowest stability values among six genes analyzed.

Table S5. Primer sequence used for real-time qPCR analysis

Gene name	Gene ID/Accession no[1]	Forward sequence (5' - 3')	Reverse sequence (5' - 3')
ZnOrco	Znev_11756	ACGCGAACGAAACCATTTTC	GAAGGACACGACGTAACCTC
ZnIR25a	Znev_19054	GGAGTTGAGATCGGAGAAGTCA	CTTGATGGCCGCGTCGTATA
ZnIR8a	Znev_18858	GAGTGTGCAACGAGGTCAGA	ATGTAAGGGATTCCTGCGCC
Znb-actin	AB915826	AGCGGGAAATCGTCCGTGAC	CAATGGTGATGACCTGCCCAT
ZnEF-1a	AB915828	GCATGCACTGTTGGCTTTTA	TTCCTCAAATCGGGTTTCAG
ZnNADH-dh	AB936819	CGGCAAGGAAGCAAATAAAG	TTGGGTTGGGGGTATCAGC
ZnRS49	KDR21989 (Znev_08151)	CATGCTTCCTACTGGCTTCC	AATTTCGGCACAGAATTTGC
ZnRPS18	KDR22651 (Znev_00110)	CTCCGTGAAGACCTGGAGAG	CGTCTTCGTGTGTTGTCCAC
ZnRPL13a	KDR22610 (Znev_00068)	CACTTCAGAGCACCAAGCAA	ACGTTTCAATGCTGCCTTTC

[1]Gene ID of *Zootermopsis nevadensis* (Terrapon et al. 2014) or DDBJ/EMBL/GenBank Accession No.
